# Humanized Chimeric Antigen Receptor (CAR) T cells

**Published:** 2021

**Authors:** Pouya Safarzadeh Kozani, Pooria Safarzadeh Kozani, Roddy S. O’Connor

**Affiliations:** 1 Department of Medical Biotechnology, Faculty of Paramedicine, Guilan University of Medical Sciences, Rasht, P.O. Box 41446/66949, Iran; 2 Student Research Committee, Medical Biotechnology Research Center, School of Nursing, Midwifery, and Paramedicine, Guilan University of Medical Sciences, Rasht, P.O. Box 41446/66949, Iran; 3 Department of Medical Biotechnology, Faculty of Medical Sciences, Tarbiat Modares University, Tehran, P.O. Box 14115/111, Iran; 4 Center for Cellular Immunotherapies, Perelman School of Medicine at the University of Pennsylvania, Philadelphia, Pennsylvania, USA; 5 Department of Pathology and Laboratory Medicine, Perelman School of Medicine of the University of Pennsylvania, Philadelphia, Pennsylvania, USA

## Commentary

In 1989, researchers proposed an intricate strategy in the field of adoptive cell therapy (ACT) [1]. Using the T-cell receptor (TCR) as a template, they replaced the coding sequence for the Vα and Vβ chains with the antigen- recognition domains from an antibody (VH and VL chains) [1]. While each format allows T cells to recognize unique antigens, the later supports T-cell activation in a major histocompatibility complex (MHC)-independent manner, such chimeric entities become known as “T-bodies” [1]. In a streamlined version of this approach, they reduced the antigen recognition moiety to a single-chain variable fragment domain (scFv) and fused it to the ζ chain of the TCR/CD3 complex [2]. Within this modular recombinant, the intracellular CD3ζ chain is sufficient to support T-cell activation following antigen engagement. From a design perspective, it explains the origin of the chimeric antigen receptors (CARs) used clinically to treat cancer. Several iterations of this approach have been developed, including very recent efforts that replace CD3ζ with CD3ε or growth factor receptor-bound protein 2 (GRB2) to permit optimal structural reconfigurations in the receptor complex and accentuate signal transduction [3]. Second-generation CARs included including “built-in” coreceptor stimulatory domains from CD28, 4-1BB and the Inducible T-cell costimulator (ICOS) to achieve complete functional competence and potency [4]. To generate CARs, scFvs are usually isolated from antibody libraries originating from an immunized host (non-human) [4–7]. There are inherent limitations to this approach as adoptive transfer can trigger an immune response characterized by the production of neutralizing antibodies against the foreign scFvs [5,6]. This limits “durable efficacy” as the administered cells are targeted for destruction and eliminated from the circulation [4–6]. Recent advances have addressed this with the aim of increasing long-term persistence and immune-surveillance following T-cell transfer.

**Figure 1:**
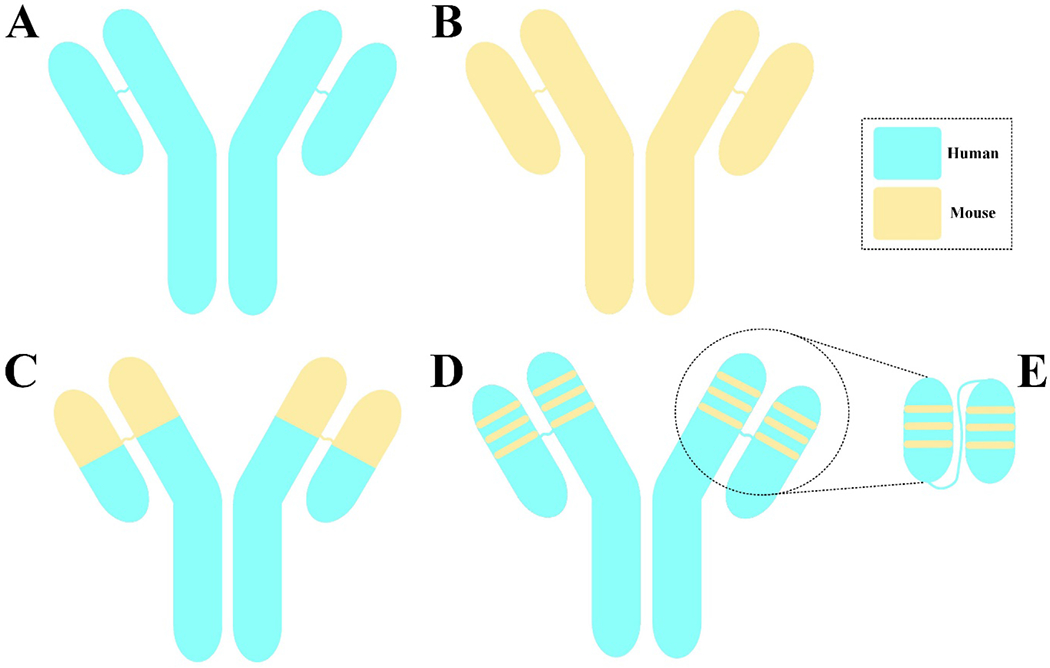
A schematic representation of a fully human **(A)**, a murine **(B)**, a chimeric **(C)**, a humanized monoclonal antibody (mAb) **(D)**, and a humanized single-chain variable fragment (scFv) **(E)**.

scFv humanization is increasingly recognized as an important design feature to optimize CAR-T cell longevity following infusion (Figure 1) [4,6]. An scFv is composed of four framework regions and three complementarity- determining regions (CDRs), which are responsible for antigen recognition [8]. CDR grafting describes a process where amino acids in the scFv framework of a murine-based CAR are substituted with those of its human counterpart [4,9]. This method is one of the most widely used approaches for the humanization of antibody fragments [9]. Given the dedicated effort to maintain high residue identification during this process, the humanized antibody fragment is expected to have similar characteristics with respect to affinity, sensitivity, and specificity as those of its native counterpart [9]. Another strategy to overcome the immunogenicity issue of animal-derived targeting moieties is to incorporate fully human antibody fragments into CAR constructs [6,10,11]; however, a limited number have been developed thus far. On this basis, scFv humanization remains the preferred option, and their therapeutic promise is currently being tested in preclinical as well as clinical settings (summarized in Table 1) within the CAR arena [12–18].

Herein, we mention some CAR-T cell products that have humanized scFvs as their targeting domains and highlight humanized monoclonal antibodies (mAbs) that achieved success in the clinics.

As of August 4, 2021, five CAR-T cell products have been granted permission by the United States food and drug administration (FDA) for medical use [19–25]. Within this therapeutic group, four CARs are designed to recognize CD19 as their target antigen (namely tisagenlecleucel, axicabtagene ciloleucel, brexucabtagene autoleucel, and lisocabtagene maraleucel) and one (namely idecabtagene vicleucel) targets B-cell maturation antigen (BCMA) [19–25]. However, all these CAR-T products rely on murine scFvs to redirect their specificity against CD19 (FMC63 scFv) as well as BCMA [19–25]. Since there have been reports regarding the immunogenicity of animal-derived targeting domains, there is room for optimization [4–6].

In 2006, Kershaw and co-investigators conducted a Phase I clinical trial to investigate the safety of folate receptor-redirected CAR-T cells in patients with metastatic ovarian cancer [26]. As reported, the administered CAR-T cells failed to react with folate receptor-expressing tumor cells in 3 out of 6 subjects (50%) which was attributed to the development of inhibitory factors in their sera [26]. Moreover, in 2011, Lamers and colleagues generated CAR-T cells against carbonic anhydrase IX (CAIX) and investigated their ability to control tumor burden in metastatic renal cell carcinoma patients [27]. Persistence issues were observed following infusion which resulted from immune reactions against the CDRs and framework regions of the CAR targeting domain. This compromised CAR-T cell-mediated antitumor responses [27]. Of note, CAR gene delivery was achieved by retroviral infection in this trial. Immune reactions against the γ-retroviral vector-encoded epitopes were also observed in 2 of the patients further exemplifying immunogenicity issues surrounding CAR transgenes and vectors used for gene transfer [27]. Finally, in a clinical trial (NCT01865617) which investigated the effectiveness of CD19-redirected CAR-T cells against B-cell acute lymphoblastic leukemia (B-ALL), Turtle et al. noted a CD8^+^ T-cell-mediated immune response against adoptively transferred cells expressing the synthetic receptor. This limited persistence of the administered CAR-T cells and increased the risk of disease relapse [28].

In 2018, Cao et al. reported the results from a clinical trial (NCT02782351) investigating the effectiveness of humanized version of the murine FMC63 antibody, included as the targeting domain of CAR-T cells in patients with R/R B-ALL (Table 1) [29]. 18 patients were enrolled in this study from which 14 did not have previous CAR-T cell therapy [29]. Among patients without previous CAR-T cell treatment, 13 (92.9%) achieved complete remission (CR) with incomplete count recovery (CRi) on day 30 [29]. Of note, CRi is defined as <5% bone marrow blasts, absence of extramedullary disease, and no recovery of peripheral blood counts independent of transfusion. Moreover, 17 patients (94.4%) experienced cytokine release syndrome (CRS) and 1 (5.5%) developed reversible neurotoxicity [29]. Of 4 patients with previous CAR-T cell therapy, 1 died on day 14 due to intracranial hemorrhage [29]. Moreover, 2 patients died after undergoing salvage therapy (one on day 145 and the other on day 169) [29]. The remaining patient was reported to be MRD-negative until day 168 [29]. These findings show that CD19-redirected CAR-T cells equipped with humanized scFvs can effectively mediate disease remission in R/R B-ALL patients even in those who have had multiple previous conventional CAR-T cell treatment [29].

In 2020, Heng and co-workers reported the results of another clinical trial (NCT02349698) investigating CAR-Ts with a humanized scFv against CD19 for the treatment of R/R B-ALL patients (Table 1) [30]. Ten patients with R/R B-ALL were enrolled in this study, all of which (100%) achieved CR, 8 patients (80%) remained CR (report published in 2020) and 6 patients (60%) had CR for more than one year and a half [30]. The researchers also reported CRS and neurotoxicity in 4 patients which was mitigated using tocilizumab, glucocorticoid, and plasma exchange [30]. They concluded that CAR-T cells equipped with humanized targeting domains demonstrate prolonged persistence leading to low rates of disease relapse [30].

In a recent clinical trial (NCT02374333), Myers et al., evaluated the antitumor response, persistence, and toxicity of CD19-redirected CAR-T cells with humanized scFvs as the targeting domain in children and young adults with B-ALL (72 patients) and B-lymphoblastic lymphoma (2 patients) (Table 1) [31]. Among these patients, 33 had previous CAR-T cell treatment with a CAR construct containing a murine scFv (FMC63) [31]. 62 patients (84%) experienced CRS and neurotoxicity was observed in 29 patients (39%) [31]. The overall response rate one month after CAR-T cell administration was 98% among the patients with no CAR-T cell treatment history and 64% among the patients with prior CAR-T cell treatment [31]. The researchers also indicated that the relapse-free survival rate at 24 months was 58% and 74% among patients with and without previous CAR-T cell treatment, respectively [31]. Collectively, these findings show that CAR-T cells with humanized targeting domains are capable of mediating durable disease remission with prolonged persistence in children and young adults with R/R B-ALL, even in patients that underwent unsuccessful treatments with CAR-T cells [31].

Several parameters are widely acclaimed to influence the effectiveness of CAR-T cell therapies including CAR-T cell quality, differentiation status, metabolic profile, and importantly CAR design [32–37]. Given the limited persistence and immunogenicity issues surrounding CAR-T cell products designed with murine-based scFvs, efforts to develop humanized versions without impairing affinity, specificity, and sensitivity might further enhance the therapeutic promise of CARs redirected against tumor antigens (Table 2). Exemplifying their therapeutic promise, several iterations of humanized CD19-specific CAR T cells are being tested in clinical trials.

## Figures and Tables

**Table 1:** A summary of clinical trials investigating CAR-T cells with humanized targeting domains.

Clinical trial identifier	Target antigen	Indication(s)	Number of patients	Start date	Completion date	Phase	Ref.
NCT02782351	CD19	R/R B-cell malignancies	50	May 2016	December 2018	I / II	[29]
NCT02349698	CD19	B-cell leukemia and lymphoma	45	December 2014	December 2023	I / II	[30]
NCT02374333	CD19	B-ALL and DLBCL	85	March 2014	November 2022	I	[38]
NCT04532268	CD19	B-ALL and B-cell NHL	72	August 2020	August 2026	Early Phase I	-

**Table 2:** A summary of humanized mAbs approved by the US FDA for the treatment of hematological malignancies.

mAb name	Trade name	Structure	Target	Indication(s)	FDA approval date	References
**Loncastuximab tesirine**	Zynlonta	Humanized IgG1 ADC	CD19	DLBCL	2021	[39]
**Belantamabmafodotin**	BLENREP	Humanized IgG1 ADC	BCMA	MM	2020	[40]
**Tafasitamab**	Monjuvi	Humanized IgG1	CD19	DLBCL	2020	[40]
**Polatuzumab vedotin**	Polivy	Humanized IgG1 ADC	CD79b	DLBCL	2019	[41]
**Mogamulizumab**	Poteligeo	Humanized IgG1	CCR4	CTCL	2018	[42]
**Inotuzumab ozogamicin**	BESPONSA	Humanized IgG4 ADC	CD22	Hematological malignancies	2017	[43]
**Elotuzumab**	Empliciti	Humanized IgGI	SLAMF7	MM	2015	[44]
**Obinutuzumab**	Gazyva	Humanized IgG1; Glycoengineered	CD20	CLL	2013	[45]
**Alemtuzumab**	MabCampath, Campath-1H; Lemtrada	Humanized IgGI	CD52	CML	2001	[46]
**Gemtuzumab ozogamicin**	Mylotarg	Humanized IgG4 ADC	CD33	AML	2000	[47]

Abbreviations: ADC: Antibody-Drug Conjugate; US FDA: United States Food and Drug Administration; DLBCL: Diffuse Large B-Cell Lymphoma; BCMA: B-Cell Maturation Antigen; MM: Multiple Myeloma; CCR4: C-C Motif Chemokine Receptor 4; CTCL: Cutaneous T-Cell Lymphoma; CLL: Chronic Lymphocytic Leukemia; CML: Chronic Myeloid Leukemia; AML: Acute Myeloid Leukemia.
